# Dual-Mode Electro-Optical Techniques for Biosensing Applications: A Review

**DOI:** 10.3390/s17092047

**Published:** 2017-09-07

**Authors:** José Juan-Colás, Steven Johnson, Thomas F. Krauss

**Affiliations:** 1 Department of Physics, University of York, York YO10 5DD, UK; thomas.krauss@york.ac.uk; 2 Department of Electronic Engineering, University of York, York YO10 5DD, UK; steven.johnson@york.ac.uk

**Keywords:** optical biosensors, electro-optical devices, label-free detection

## Abstract

The monitoring of biomolecular interactions is a key requirement for the study of complex biological processes and the diagnosis of disease. Technologies that are capable of providing label-free, real-time insight into these interactions are of great value for the scientific and clinical communities. Greater understanding of biomolecular interactions alongside increased detection accuracy can be achieved using technology that can provide parallel information about multiple parameters of a single biomolecular process. For example, electro-optical techniques combine optical and electrochemical information to provide more accurate and detailed measurements that provide unique insights into molecular structure and function. Here, we present a comparison of the main methods for electro-optical biosensing, namely, electrochemical surface plasmon resonance (EC-SPR), electrochemical optical waveguide lightmode spectroscopy (EC-OWLS), and the recently reported silicon-based electrophotonic approach. The comparison considers different application spaces, such as the detection of low concentrations of biomolecules, integration, the tailoring of light-matter interaction for the understanding of biomolecular processes, and 2D imaging of biointeractions on a surface.

## 1. Introduction

The diagnosis and treatment of many diseases relies on our ability to detect disease-specific protein biomarkers [1] and to quantify biomarker concentration, which can be as low fg/mL [2,3,4,5,6]. Although affinity biosensors have been used for protein detection for over five decades [7], they are still some of the most important diagnostic tools for protein biomarker quantification. Affinity biosensors typically consist of a surface-immobilised receptor, often an antibody, which binds to a specific biomarker with high affinity and high selectivity. The formation of the antibody-antigen complex is subsequently detected through the addition of a label, typically enzymatic, fluorescent or radioactive, which translates the binding event into a quantifiable signal. Despite the widespread use of these laboratory techniques, the reliance on an additional labelling step not only limits the throughput but can also, in some cases, interfere with the binding process [8]. In order to address these limitations, a range of novel, label-free biomarker detection techniques have been demonstrated which allow for simple and fast detection of biomarkers in their native conformation and at relevant clinical concentration levels, without the need for an additional labelling step [9].

Surface affinity label-free biosensors consist of two key elements: (a) a surface-immobilized bioreceptor which has high affinity, specificity and selectivity for the target biomarker, and; (b) a transduction element which translates the biochemical binding event between the target and the bioreceptor into a physical, measurable signal. Several strategies have been developed for transducing this biomolecular binding event, including acoustic [10,11], electrical [12,13,14] or optical [15,16,17,18] methods. These biosensors are sensitive to a single parameter, for example the change in mass, charge or refractive index that occurs following binding, and are thus “single-domain” techniques. Single-domain electrochemical biosensors have received significant attention [12,14], driven largely by the opportunity to exploit technological developments in the electronics industry. More recently, advances in optics and photonics have led to the introduction of optical transduction techniques with similar limits of detection [15,16,17,18] to their electrochemical counterparts. Optical techniques offer unique advantages, such as immunity to electromagnetic interferences, self-referencing by tracking optical resonances rather than continuously measuring current intensities and the availability of unique optical absorption and spectral fingerprinting features available with for example, Raman spectroscopy. Furthermore, optical techniques are non-contact, thereby avoiding degradation and corrosion effects that often occur with electrochemical sensors [19], as well as allowing parallel readout capability. These capabilities underpin the potential for portable instruments that are capable of providing rapid and accurate diagnosis at point-of-care. However, optical technologies require integration with an optical source, which increases the complexity of the system compared to their electrical counterparts, especially as the source often needs to be precisely aligned with the optical input. Similarly, bulk and expensive readout instrumentation (e.g., a spectrometer) is often required to extract the desired information from the sensor.

**Figure 1:**
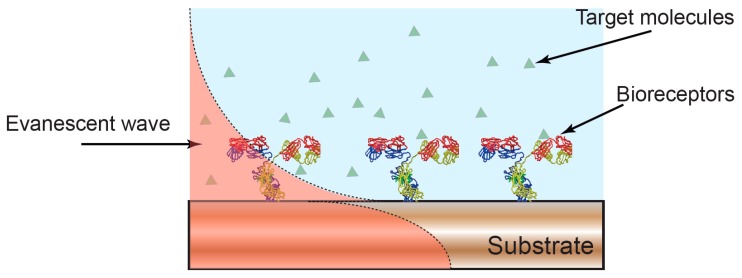
Optical biosensors based on evanescent wave detection. The properties of the optical mode confined in a photonic substrate are sensitive to the overlap between the evanescent field and the surrounding environment. A change in the local refractive index, for example, due to formation of an antibody-antigen complex, leads to a change in the properties of the confined optical mode and to a quantifiable readout of molecular binding.

Typically, optical transduction in photonic biosensors is enabled by the sensitivity of an optical surface mode to changes in the local refractive index. By sensitizing the surface to a specific molecule, for example by immobilization of a specific bioreceptor, the local change in refractive index that occurs following a biochemical event, is transduced into the properties of the mode to provide a quantifiable readout [16,20,21,22] (Figure 1). This phenomenon, called evanescent-wave sensing [16,20,21,22], is the most common principle exploited for optical sensing [15,17,18,23]. Depending on the particular optical technique, the changes in the properties of the optical mode can led to a shift in resonance wavelength [24,25,26], interference pattern [18,27] or coupling angle [15].

A wide range of optical techniques that provide accurate, fast and reliable single-domain analysis of biochemical events occurring at the surface of the sensor have been demonstrated [15,16,17,18,23,28]. Recently, multi-domain methods have been gaining in popularity, as they can simultaneously probe different properties of a biochemical reaction and thereby increase the amount of information that can be extracted from the system. For example, the combination of acoustic and electrochemical sensing has been shown to provide real-time information about binding kinetics, molecular conformation and electrochemical properties of biomolecules [29]. Similarly, the combination of optical and electrochemical sensing can provide unique insight into dynamic structural and electrochemical properties, for example, to investigate enzyme activity [30], or to monitor electrostatic adsorption of polymer films onto surfaces [31]. Critically, the combined electro-optical measurements employed in these examples provides information that would not be available with the respective techniques in isolation. They facilitate the acquisition of other characteristics that are not acquired from optical interrogation alone, such as electrical impedance or charge. Furthermore, the integration of multiple measurement modalities not only provides access to complementary information, but also provides the opportunity to regulate and monitor the local surface chemistry in situ. For example, the spatial confinement provided by electrochemical processes can be used to control the functionalization of individual sensors within an optical biosensor array [32,33]. However, they require some degree of adaption from their single-domain versions, critically the need to incorporate electrochemical control over the sensor surface. In some cases, the measurement system needs to be adapted to be able to perform such electrochemical control, which, depending on the technology, can be achieved by coating the sensing layer of the system with electrically and optically compatible layers [31], or by adapting the electrical characteristics of the sensing layer so that it can perform measurements in both domains [32,33].

In this review, we provide an overview of the most developed electro-optical techniques for (bio)sensing applications, namely, electrochemical surface plasmon resonance (EC-SPR), electrochemical optical waveguide lightmode spectroscopy (EC-OWLS), and electrophotonic silicon biosensing. Individual and comprehensive reviews of the single-domain versions of these techniques (i.e., without electrochemical interrogation) can be found in [15,16,17,18,23].

## 2. Electro-Optical Multi-Domain Techniques

### 2.1. Electrochemical Surface Plasmon Resonance (EC-SPR)

**Figure 2:**
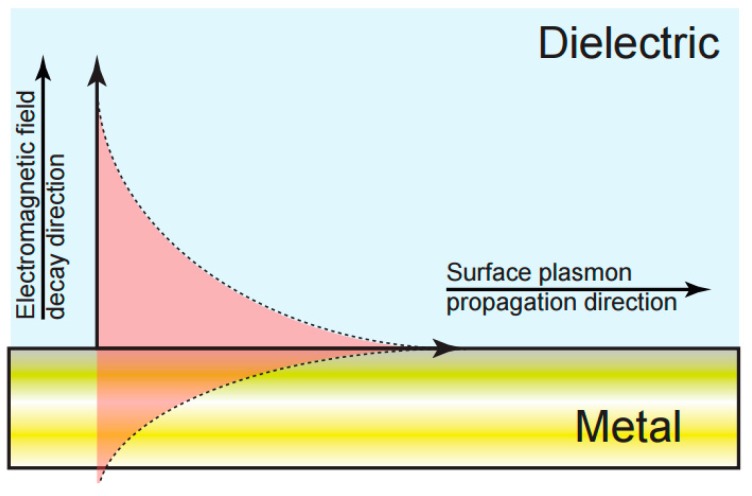
Schematic of surface plasmon resonance (SPR) field distribution at a metal-dielectric interface. The overlap of the evanescent field with the dielectric phase of the interface provides a direct measurement of changes in the local refractive index.

The most widespread, mature and well-known label-free optical biosensor technology is surface plasmon resonance (SPR) [15], first commercialised by Biacore^TM^. SPR uses surface plasmon waves to directly measure the change in local refractive index induced by biomolecular interactions occurring on a metal surface [15] (Figure 2). Surface plasmons are charge density oscillations that can be excited optically at an interface between a dielectric and a metal by matching the wavevector of the incoming light with that of the surface plasmon, typically by controlling the angle of the incoming light beam. A change in the wavevector of the plasmon, for example, caused by the binding of an antigen to a surface immobilized antibody, leads to a shift in the resonance angle which provides a label-free and quantifiable measurement of the mass adsorbed on the surface. This technique has the capability of detecting extremely low concentrations of molecules with limits of detection in aqueous buffer of 0.74 fg/mL [5] and 13 ng/mL [34] in a complex sample medium.

A number of commercial instruments based on SPR have already been developed, included those marketed by Biacore (a division of GE Healthcare), GWC Technologies, IBIS Technologies, Toyobo, GenOptics, SensiQ and Bio-rad. Since SPR was first demonstrated three decades ago, a range of sub-techniques have emerged, including localised surface plasmon resonance (LSPR) [35] and long-range surface plasmon resonance (LR-SPR) [36], both of which have been used in sensing and imaging applications. The best limits of detection (typically in the low ng/mL to high fg/mL range for protein interactions [15,18,37,38,39]) are obtained when interrogating the sensor with angular or wavelength spectroscopy, and up to 10 measurements can be performed simultaneously [40].

**Figure 3:**
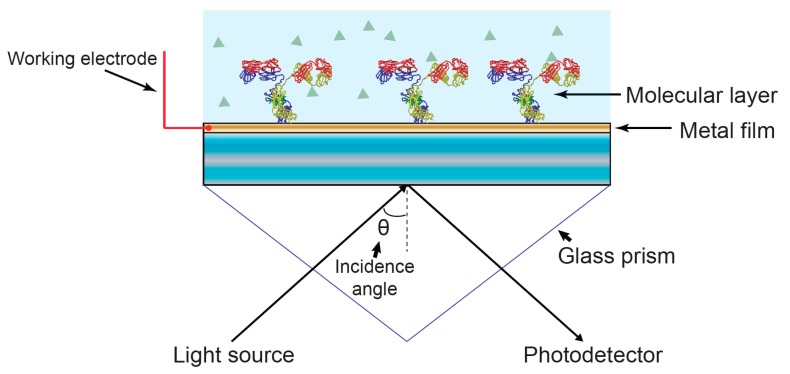
Schematic diagram of electrochemical-SPR (EC-SPR). The gold substrate that carries the optical surface mode is simultaneously used as the working electrode, allowing simultaneous optical and electrochemical interrogation. Typically, the fluidic manifold also includes counter and reference electrodes to form a complete three-electrode electrochemical cell.

As an extension of SPR, electrochemical-SPR (EC-SPR) (Figure 3) was developed with the aim of probing extremely small changes in electrostatic fields at electrodes surfaces [41], and to study the structure and activity of redox active systems [42]. In such systems, a three-electrode assembly is integrated into the SPR in order to the study the interaction between electrical energy and chemical change. In the three-electrode electrochemical cell, a potential difference is applied between the working electrode (gold film where the surface plasmon is excited, Figure 3) and counter electrode (typically a Pt wire), while the current flowing through the circuit as a result of an electrochemical reaction is measured. The potential required to drive the working electrodes is supplied relative to the reference electrode that presents a potential relative to the solution which is known and stable.

In EC-SPR systems, the working electrode is used to control the potential and interfacial electric fields. The potential of the working electrode is commonly cycled between two potentials at a constant rate (i.e., known as cyclic voltammetry (CV)). In parallel, the current is continuously monitored through the counter electrode. The resulting current contains two components; a non-faradaic component, due to charging of the interfacial capacitance, and a faradaic component associated with an electrochemical reaction. This is exploited in EC-SPR to greatly extend the application space available to SPR, enabling SPR to be applied to the study of enzymatic processes [43], anodic stripping [44,45], potential-controlled molecular adsorption, charge transfer processes [42,46], and the characterization of electrochemical DNA sensors [47].

More recently, the combination of SPR with electrochemical impedance spectroscopy (EIS) [48] has been shown to lead to a 1.35-fold increase in specificity of molecular binding (due to reduced sensitivity to the bulk refractive index and nonspecific binding effects). In this case, however, the electrochemical measurement is based on the application of a small sinusoidal potential superimposed on a direct current (DC) bias where the current is measured as a function of frequency, enabling measurement of the electrochemical impedance.

### 2.2. Electrochemical Optical Waveguide Lightmode Spectroscopy (EC-OWLS)

**Figure 4:**
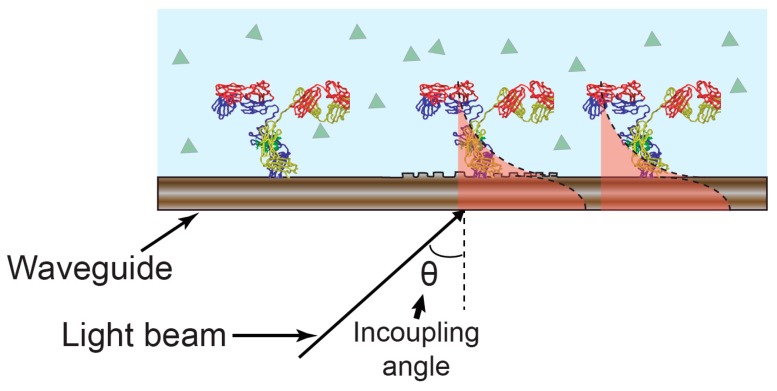
Optical waveguide lightmode spectroscopy (OWLS) sensing mechanism. The incoupling angle varies as a function of the refractive index as experienced by the guided optical mode.

Electrochemical optical waveguide lightmode spectroscopy (EC-OWLS) combines evanescent field optical sensing with the electrochemical control of surface adsorption processes. Here, electrochemical functionality is provided through the incorporation of an optically and electrochemically compatible layer on the top of an optical structure. Optical waveguide lightmode spectroscopy (OWLS) was first presented in the mid-1980s as a label-free technique for studying adsorption, desorption, adhesion, and biospecific binding processes at biomaterial surfaces such as silicon titanium oxide (Si(Ti)O_2_) [49,50]. In this technique, a grating is used to couple light into a waveguide at two characteristic angular values which relate to the transverse electric (TE) and transverse magnetic (TM) modes. This pair of coupling angles varies as a function of the refractive index at the solution phase of the waveguide-solution interface. By monitoring the coupling angles, the properties of the adsorbed layer [51] can thus be determined. Figure 4 illustrates the sensing mechanism.

The OWLS technique has since been demonstrated for monitoring environmental pollution, the assembly of lipid layers, and for monitoring protein-DNA interactions [52]. The sensitivity of OWLS is lower than that of SPR because of the weaker light-matter interaction at the interface. As a result, limits of detection in the regime of 100s of ng/mL have been reported [53]. The technology was brought to the market by the company OWLS (Budapest, Hungary), which commercialized the system OWLS 210^®^ in 2002.

**Figure 5:**
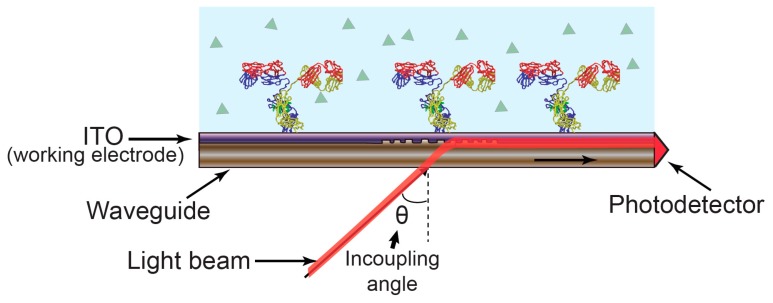
Electrochemical optical waveguide lightmode spectroscopy (EC-OWLS). The angle of light coupled into the waveguide by the grating depends on the refractive index of the molecules attached to the sensor surface while the indium tin oxide (ITO) provides an electrically conductive layer to support electrochemistry.

The incorporation of electrochemical interrogation with OWLS was first reported in 2002. Electrochemical functionality was included by the deposition of a thin layer of indium tin oxide (ITO) working electrode on the waveguide surface, while the flow cell contains counter and reference electrodes to provide a three-electrode electrochemical configuration. The ITO serves both as a high-refractive-index waveguide and as a conductive working electrode, thereby addressing both the optical and the electrochemical requirements [31,54] (Figure 5).

EC-OWLS was first demonstrated for monitoring and controlling the adsorption of an electrostatically interacting polymer as a function of the applied potential, which provided insight into the adsorption kinetics of the charged moieties [31,54,55,56]. This technique has been used to simultaneously provide control and monitoring of adsorption kinetics in situ, monitor the adsorbed mass of charge molecules, and to study the reversibility/irreversibility of adsorption processes. It has been also employed in many diverse applications, such as for characterising layer-by-layer assembly [57] of thin film polyelectrolyte multilayers [58], studying the viability of cells under the influence of applied currents [59], surface modifications [60,61], molecular interactions [58], and the survival of lactic acid bacteria [62]. As with EC-SPR, the technique has also been demonstrated as compatible with EIS for monitoring the growth of lipid bilayers [63].

### 2.3. Electrophotonic Silicon Biosensing

Diagnostic technology based on silicon has now become one of the most promising optical sensing modalities. Silicon photonic sensors have emerged from the silicon photonic technology developed originally for the communications industry. The use of silicon not only provides the high index contrast that is critical for the realization of high-density sensor arrays, but also enables the use of CMOS fabrication technology to provide high-quality, low-cost fabrication of portable, photonic integrated circuits (PICs) [64].

Silicon photonic biosensors exploit the changes in the propagation constant of optical modes caused by the presence of a surface-immobilized biomolecular layer. A number of highly sensitive optical techniques can be used to monitor this optical shift, for example, interferometry caused by the difference in propagation length or the shift in resonance caused by the change in refractive index due to binding, both leading to a quantifiable signal that is proportional to the amount of biological material present on the sensor surface [13,17,18,22,23,65].

Mach-Zehnder interferometers (MZI), which translate the phase difference between two light waves into a measurable intensity difference, are a notable example of interferometric measurement modality. Similarly, bimodal waveguide interferometers translate the difference in propagation constant between different order modes into a change in intensity. These types of interferometric measurements have demonstrated sensitivities for protein concentration in the range of pg/mL [23,66]. Similar sensitivity can be achieved with resonant cavity-based devices, such as photonic crystals (PhC) [28] and ring resonators [67], and detection limits in the low pg/mL range [24] have been demonstrated. Silicon photonics has now sufficiently matured to the extent that biosensors are already available commercially. For example, the Maverick^®^ system (Genalyte, San Diego, CA, USA) contains a highly multiplexed system featuring 128 independent ring resonator sensors. This represents the highest degree of multiplexing on the market [68,69] (e.g., more than 10 times the number of parallel sensors compared to SPR [40]).

**Figure 6:**
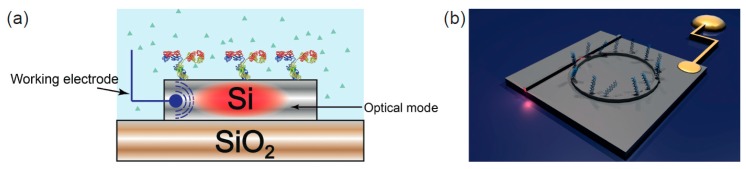
Electrophotonic silicon biosensing. (**a**) Cross section of an electrophotonic silicon biosensor. The electrochemical functionality is provided by doping the surface of the silicon waveguide layer; (**b**) Sketch of an electrophotonic silicon sensor based on a ring resonator structure.

Even though the microelectronics revolution is based on silicon technology, the opportunities for integrating silicon photonics with electrochemical sensing has so far been surprisingly ignored. By applying carefully controlled doping of silicon, we have recently addressed this oversight and have added electrochemical functionality to the silicon photonic toolkit. This novel sensing modality, which we refer to as electro-photonic silicon biosensing, has been demonstrated using optical ring resonators on a silicon-on-insulator (SOI) substrate [1,2] (Figure 6).

In order to combine the electrochemical and the optical modalities, the electrical properties of the silicon on insulator (SOI) substrate were modified by controlling the doping profile of the silicon layer. Specifically, by only doping the first 10–20 nm of the silicon layer, we showed it was possible to create a silicon layer that was sufficiently conductive to support electrochemistry, yet thin enough to have little impact on the photonic performance of the sensor [32,33]. The electrochemical control over the sensor surface was then achieved by fabrication of an ohmic contact to the silicon layer [32,33], which ensured efficient electron tunnelling in and out from the silicon surface into the supporting solution (as required for electrochemical measurements). Similar to EC-SPR and in EC-OWLS, a three-electrode electrochemical assembly was incorporated to the system to perform electrochemical interrogation (capable of performing both CV and EIS).

We employed this technology to monitor molecular structure and activity of electrochemical reactions occurring at the silicon surface, for example, the surface coverage of adsorbed electroactive monolayers or to monitor electrochemical reactions in situ. Moreover, the combination of the two sensing modalities was further exploited to spatially control the surface chemistry. This was achieved through the electroreduction of diazonium ions [32,33]. Since the electroreduction of these moieties is highly localised (i.e., occurs only where the electrical potential is applied), nanometre-scale spatial resolution can be achieved for creating high-density arrays [70,71,72]. In this way, using diazonium salts moieties with different functional groups, we were able to selectively and spatially direct the immobilisation of different capture molecules onto different sensors within an array, here single-stranded DNA oligonucleotides, all inside one microfluidic channel.

We note, while the electro-photonic technology was demonstrated using the well-established ring resonator architecture, the concepts are generic and could be implemented on a wide range of alternative photonic sensors, such as photonic crystals [16,28], and guided mode resonances [73,74].

### 2.4. Comparison between Optical Dual-Mode Sensing Techniques

We now compare the capabilities and limitations of the various dual-mode sensing modalities in light of suitable applications, such as their intrinsic limits of detection, their integration capabilities and their abilities to help with the understanding of biomolecular processes. We have chosen not to compare the three techniques using a single figure of merit since this most appropriate choice of metric depends on the application. Instead, we compare three technologies via three performance measures, namely the intrinsic detection limit, the amenability for future technology integration, and the ability to control and thus optimise the light-matter interaction.

#### 2.4.1. Intrinsic Detection Limit

The first question to ask is whether the addition of the electrical/electrochemical capability impacts on the optical performance of the sensor. For EC-OWLS, the addition of the ITO layer on to the dielectric waveguide increases the optical loss, and therefore reduces the optical performance (typical optical transmission of an 80 nm-thick ITO layer is 85% at a wavelength of 850 nm [75,76]). For the silicon electro-photonic approach, we were able to show that the impact on the optical performance is minimal by only doping the very surface of the sensor, that is, the first 10–20 nm. In this specific example, the Q-factor of the optical resonance decreased from Q ≈ 60 k to Q ≈ 50 k, with a commensurate reduction in the limit of detection.

By comparison, EC-SPR naturally uses a highly conductive metal surface, and therefore requires no optimisation of the conductivity of the surface to allow electrochemical control [77]. SPR, in fact, does not rely on the quality factor of the resonance, but instead on the high sensitivity of the plasmon mode. As a result, there is no an intrinsic degradation of the SPR sensitivity when electrical functionality is added. Therefore, the limits of detection achieved with SPR (e.g., fg/mL, as mentioned in Section 2.1) are similar to those achieved in EC-SPR [47].

#### 2.4.2. Integration

The literature contains many examples of compact silicon photonic devices (without electrochemical interrogation) for biosensing applications, including ring resonators [25], PhCs [78] or MZIs [79]. A notable advantage of silicon photonic technology over OWLS and SPR is the potential to benefit from the highly developed mass-fabrication approaches used in the microelectronics industry and the ability to integrate light sources and detection systems into a complete and fully operative lab-on-chip (LOC) device [18]. This integration can be either (a) monolithic, in which the complete system is integrated on a single chip, or (b) hybrid, where the required functionality is implemented in multiple components that are subsequently combined on a single carrier. Monolithic integration is ultimately preferred due to higher performance and lower cost, but hybrid approaches are currently more common because they are currently easier to realise. For example, attempts to fabricate a complete and autonomous LOC device with a total component cost of $10 (US Dollar) based on a PhC sensor [80] are already underway. There are also demonstrations of LOC devices where a spectrometer is integrated on-chip to further increase the detection sensitivity [81,82].

Integration can also be exploited to create very high density arrays of photonic biosensors [64]. Additionally, the electro-photonic technology provides spatial control of the surface chemistry with a resolution which is orders of magnitude higher than that achieved with conventional microfluidic channels [69], inkjet [83] and ink-dot printing approaches. This high resolution feature is fundamental for the design of very high density biosensor arrays by allowing the selective functionalisation of individual optical sensors within an integrated sensor array. Such array-format diagnostics underpin the move towards personalized medicine, where the highly multiplexed and sensitive detection of multiple biomarkers is required. Ultimately, integrated arrays of electro-photonic biosensors could be realised that enable the monitoring of tens if not hundreds of biomarkers in a single microfluidic channel (representing a much higher degree of multiplexing than that achieved with the Maverick^TM^ system [69] or EC-SPR [40]), while still requiring only small volumes of a clinical sample. Clearly, this high integration density is also of interest for drug screening and drug development.

#### 2.4.3. Tailoring of Light-Matter Interaction for the Understanding of Biomolecular Processes

In EC-SPR, the light matter interaction is fundamentally fixed and restrained as the excitation of the surface plasmon is limited by its physical properties. For example, a surface plasmon is TM-polarised [15,84], with its k-vector orthogonal to the propagation direction. Therefore, the operation in SPR is limited to TM polarisation only.

**Figure 7:**
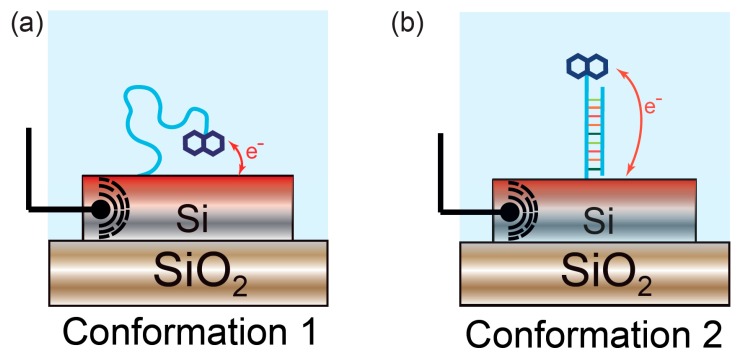
Monitoring of conformational changes with electro-photonic silicon biosensing. (**a**) Single-stranded DNA is immobilised on the sensor surface. A redox active probe linked to the DNA oligonucleotide enables electrochemical measurement of DNA hybridisation; (**b**) Upon binding to its complementary strand, the surface-immobilised DNA strand undergoes a conformational change, displacing the electrochemical probe away from the surface. This modifies the thermodynamics of the electron-transfer process, hence exhibiting changes in the electrochemical response. This conformational change can simultaneously be monitored in real-time and quantified using the underlying silicon photonic sensor.

In contrast, an advantage of both EC-OWLS and electro-photonic silicon sensing is that they allow the operation to be tailored in order to interrogate a surface molecular layer in a multimode fashion or with different depth sensitivity. For instance, both TE and TM modes can be excited in EC-OWLS, enabling information about both the refractive index and thickness of the absorbed film to be extracted [85]. Similarly, the light-matter interaction can be engineered in integrated photonic devices and a ring resonator biosensor capable of supporting both TE and TM modes in parallel has been demonstrated, which was able to monitor changes in molecular conformation in real-time [86]. This dual optical mode sensor is fully compatible with the electro-photonic technology. One can therefore envisage a device capable of determining molecular conformation with parallel electrochemical interrogation (Figure 7). The ability to control the light matter interaction can also be exploited to increase detection sensitivity. For example, slot-waveguide-based devices [87,88] and slot PhCs [89,90] confine the optical mode in an air cavity, thus increasing the overlap of the evanescent field with the (bio)molecules.

Integrated photonic devices can also be engineered to control the penetration depth of the optical mode. This degree of freedom can be exploited in order to provide different depth resolution, or to probe different regions within the surface-bound molecules. For instance, one can envisage an array of sensors in which each sensor element exhibits a different penetration depth. Devices with a short penetration depth will interact more strongly with surface-bound molecules as they are confined to the sensor surface; while, in contrast, sensors with longer penetration depth than the length of the surface-bound molecules will sense changes in the supporting solution, hence providing a higher sensitivity towards changes in the solution.

#### 2.4.4. 2D Imaging of Biointeractions on a Surface

In contrast to EC-OWLS, both EC-SPR and the electro-photonic approach can also be used to create 2D images of molecular interactions on a surface. With EC-SPR, this imaging capability is possible as plasmon resonances can be highly localized; however, the sensitivity achieved with this approach is typically five times lower than that achieved with standard SPR [40,91,92,93,94]. Finally, another capability of integrated optics is the label-free imaging of biomolecular interactions through PhCs based on guided mode resonant (GMR) surfaces [95,96,97]. These sensors are illuminated with out-of-plane light which gives them the unique advantage of easy integration with a standard microscope, hence reducing the complexity compared to EC-SPR imaging systems. These advantages have yet to be developed further to incorporate electrochemical interrogation, but the potential is clear for multi-parametric imaging of biological entities such as cells and bacteria.

#### 2.4.5. Comparison Summary

Table 1 summarizes the main points discussed here. For each technology reviewed in this paper, the table is then categorized in the five different subsections: degradation on the intrinsic detection limit by EC integration; integration with other systems for LOC devices; tailoring of light-matter interaction; 2D imaging, and; commercial availability of the electrochemical version.

## 3. Conclusions

A wide range of label-free biosensors have been demonstrated that are able to detect the presence of target molecules with high sensitivity and specificity, and that translate the presence of molecules into a measurable and quantifiable signal. To date, the majority of biosensors have exploited a single transduction domain. However, the information that can be retrieved from parallel, complementary measurements performed in multiple domains can provide much deeper understanding about a biomolecular process. Due to the complementary and uncoupled information between optical and electrical sensing domains, technologies that exploit the combination of the two are becoming more widespread for applications in, for instance, fundamental research of biomolecular interactions or the study of electroactive molecules. This combination of sensing modalities also provides additional insight into chemical activity which is not available with photonic detection alone, opening new sensing modalities. Accordingly, commercialized devices that make use of these techniques have been introduced in the market, EC-SPR being the most widely employed. The diverse field of biosensing requires a versatile technology which can be tailored to the application. Optical technologies have shown their advantages against their electrical counterparts (immunity to electromagnetic interferences, self-referencing, spectral fingerprinting, etc.), and therefore they are better placed to address this challenge; while despite slightly reduced sensitivity, the design flexibility associated with the electro-photonic approach is emerging as a potential technology to meet this need. It outperforms EC-SPR and EC-OWLS in terms of ease of integration with other systems and the tailoring of light-matter interaction, while still having the potential to detect biomolecules at relevant clinical concentrations. In addition, in both academia and the private sector, there is a huge research effort in developing completely autonomous, easy-to-use lab-on-a-chip devices which can measure multiple biomarkers in parallel. Again, and due to its ease of integration with other systems, its ability to functionalize sensors in an array at high spatial resolution and advantages against the electrochemical based sensors, the electro-photonic approach seems better positioned to achieve this goal. Finally, given the degree of freedom in the tailoring and optimization of the light-matter interaction through the control of the photonic sensor, the electro-photonic approach seems again to be best suited for applications which study biological processes such as complex conformational changes or sensing inside eukaryotic cells.

## Figures and Tables

**Table 1:** Comparison between EC-SPR, EC-OWLS and electro-photonic sensing. Lab-on-chip (LOC).

Technology	Degradation on the Intrinsic Detection Limit by EC Integration	Integration for LOC Devices	Tailoring of Light-Matter Interaction	2D Imaging	Commercial Availability
EC-SPR	Not degraded [3]	Complex [4]	Not possible [5]	~5 times lower resolution than SPR [4,6,7,8,9]	Yes
EC-OWLS	Degraded [10,11]	Complex, no reports found	Achievable to some extend [10]	Capable, but no reports found	Yes
Electro-photonic approach	Minimally degraded [1,2]	Easy	Complete freedom [1,2]	Capable with minimal degradation	No
